# Development and validation of a multidimensional organizational dehumanization scale: Evidence from higher education

**DOI:** 10.1371/journal.pone.0351254

**Published:** 2026-07-06

**Authors:** Semin Kazazoglu, Dilruba Sahin, Cem Oktay Guzeller, Dilek Ilhan-Findikoglu, Fuat Findikoglu

**Affiliations:** 1 Department of Foreign Languages Education, Faculty of Education, Yildiz Technical University, Güngören, İstanbul, Türkiye; 2 Department of Primary Education, Faculty of Education, Biruni University, Zeytinburnu, İstanbul, Türkiye; 3 Department of Psychology, Faculty of Science and Letters, İstanbul Arel University, Zeytinburnu, İstanbul, Türkiye; 4 Department of Educational Sciences, Faculty of Education, Yildiz Technical University, Güngören, İstanbul, Türkiye; Gelisim University: Istanbul Gelisim Universitesi, TÜRKIYE

## Abstract

Organizational dehumanization has become a salient concern in contemporary academic work, particularly in higher education systems shaped by managerial and metric-driven governance. This study aimed to develop and validate a psychometrically robust instrument to assess academics’ perceived organizational dehumanization. Using two independent samples of academics in Türkiye (EFA sample: n = 318; CFA sample: n = 263; total N = 581), we examined the scale’s factor structure, reliability, and validity evidence. Exploratory and confirmatory factor analyses supported a two-factor model reflecting Devaluation and Instrumentality, yielding a final 34-item scale. The model demonstrated acceptable-to-strong fit indices (χ²/df = 2.28; CFI = .99; NFI = .97; RMSEA = .074; SRMR = .045), high internal consistency (α = .93−.96; overall α = .96), and evidence for discriminant validity, with mixed evidence for convergent validity at the subdimension level. Overall, the findings suggest that the proposed scale provides a reliable, multidimensional measure of academics' organizational dehumanization perceptions, validated in a higher education sample, and offers a tool for future research and institutional diagnostics in university contexts.

## Introduction

Organizational dehumanization refers to employees’ perceptions that their organization treats them as objects, tools, or interchangeable resources rather than as full human beings with dignity, agency, and emotional subjectivity. Conceptually, the construct is grounded in dehumanization theory, which distinguishes mechanistic forms, seeing individuals as machine-like and instrumental, and animalistic forms, denying civility and moral worth [1,2]. Within organizational settings, mechanistic dehumanization, characterized by objectification and instrumentalization, has been identified as the predominant form [3,4].

Growing research suggests that perceived organizational dehumanization is associated with a range of negative employee outcomes. For example, lower perceived organizational support has been linked to stronger dehumanization perceptions, which in turn relate to reduced well-being [5]. Dehumanization has also been associated with work stress and lower work engagement [6], workplace ostracism experiences [7], abusive supervision [8], deviant or counterproductive behaviors [9], and affective commitment [10]. Additional studies indicate that organizational dehumanization is related to reduced creative performance [11] and increased opportunistic behavior [12]. A recent study further showed that organizational dehumanization partially mediates the relationship between job insecurity and emotional exhaustion, and that employee resilience moderates the relationship between dehumanization and exhaustion [13]. Together, these findings indicate that perceiving oneself as treated in an instrumental or object-like manner may undermine both employee well-being and organizational functioning.

In addition to these consequences, recent research suggests that contemporary organizational practices may themselves intensify such perceptions. Furthermore, studies on AI and digital transformation in organizations highlight that AI-enabled monitoring, automation, and metric-based evaluation may contribute to dehumanizing workplace conditions by weakening fairness perceptions, autonomy, dignity, and human connection [14–16]. These risks may be especially salient in knowledge-intensive and performance-driven sectors, where work is increasingly governed by quantifiable metrics and managerial control. Higher education represents a particularly relevant context in this regard, as universities have progressively adopted governance systems that prioritize measurable outputs such as publication counts, rankings, and external funding indicators. Scholars argue that such managerial regimes can foster precarity and a sense of disposability among academics while reducing recognition of them as whole persons rather than merely as units of productivity [17,18]. Relatedly, research on everyday academic life in neoliberal universities suggests that these performance pressures may also unsettle academic identities and weaken senses of belonging, generating experiences of restlessness as individuals strive to conform to standardized expectations of “excellence” [19]. Taken together, these structural conditions indicate that academics may be particularly vulnerable to interpreting organizational practices as dehumanizing.

Despite growing empirical attention, the measurement of organizational dehumanization remains relatively limited. The most widely used instrument is the unidimensional Organizational Dehumanization Scale developed by Caesens et al. [5], with a validated short form proposed by Lagios et al. [20]. Although these measures show strong psychometric performance, their unidimensional structure and general organizational framing may provide limited coverage of how dehumanization is enacted through distinct, yet related, processes. This may be particularly consequential in knowledge-intensive and high-precarity settings such as higher education, where academics may experience dehumanization both as diminished interpersonal recognition and as being treated as an instrumental, replaceable resource [4]. Accordingly, existing measures rarely differentiate between perceptions of devaluation and instrumentality, which may constrain more nuanced assessment in academic work contexts.

To address these limitations, the present study develops and validates a ''Multidimensional Organizational Dehumanization Scale'' and evaluates its psychometric properties in a higher education sample. Grounded in mechanistic dehumanization and organizational justice perspectives, we conceptualize perceived organizational dehumanization as comprising two related dimensions: Devaluation, reflecting diminished recognition and relational worth, and Instrumentality, reflecting objectification and perceptions of replaceability. We evaluate the scale using exploratory and confirmatory factor analyses, internal consistency estimates, and validity evidence. By providing a multidimensional instrument validated among academics, this study enables a more differentiated assessment of academics' dehumanization perceptions and supports institutional diagnostics and interventions aimed at fostering more humane academic workplaces.

## Literature review

### Organizational dehumanization: A review of antecedents and consequences

Organizational dehumanization is the perception that one's organization treats employees as mere tools, objects, or interchangeable instruments for achieving institutional goals, denying their subjectivity, emotions, individuality, and human qualities [5]. This perception may arise in work contexts marked by instrumental treatment and power asymmetries, in which employees are valued primarily for their functional utility, and may come to feel objectified or reduced to their usefulness [21]. Organizational dehumanization primarily aligns with mechanistic dehumanization [1], involving the denial of human nature attributes such as warmth, emotional depth, agency, and individuality, portraying employees as cold, rigid entities.

Haslam [1] further distinguishes animalistic dehumanization, which entails the denial of uniquely human characteristics such as morality, civility, and rational thought, and is typically directed toward essentialized outgroups in contexts where the ingroup is represented communally. Animalistic dehumanization is frequently associated with feelings of contempt and disgust, which signal an implicit hierarchical comparison, and manifests in attributing others’ actions to basic desires and wants rather than cognitive states [1,2]. While animalistic dehumanization has traditionally been examined in intergroup contexts, organizational contexts may trigger both mechanistic and animalistic framings, for example through professional, departmental, rank-based, or social group stereotypes [3]. Although both mechanistic and animalistic dehumanization can occur in workplace settings, mechanistic dehumanization is generally considered more prevalent in organizational hierarchies and efficiency-driven contexts, as it directly denies the emotional depth, autonomy, and individuality that distinguish human beings from instruments or machines [3].

Organizational dehumanization does not emerge randomly but is triggered by specific structural, interpersonal, and environmental conditions within organizations. At the organizational level, low perceived organizational support, violations of procedural justice, and the proliferation of red tape, defined as unnecessary, ineffective, and burdensome organizational rules, directly exacerbate perceptions of being dehumanized [5,22,23]. Organizational cultures emphasizing efficiency and productivity at the expense of employee dignity and meaningfulness further reinforce mechanistic framings of workers as interchangeable units [22,23]. At the supervisory and interpersonal level, abusive supervision [8], poor leader-member exchange quality [24], and workplace ostracism from both supervisors and coworkers [7] serve as critical triggers, signaling to employees that they lack value as human beings within the organizational system. Job-related conditions including low autonomy, repetitive and fragmented work, and technological dependence contribute to mechanistic dehumanization by systematically denying employees agency and meaningful involvement in their work [22]. Similarly, organizational powerlessness, which is defined as the perception that one lacks voice and influence in decision-making processes, amplifies the experience of being treated as an instrument rather than an autonomous agent [23]. These antecedent conditions collectively create structural and relational contexts in which employees develop perceptions of being instrumentalized and reduced human worth.

The pathway through which organizational dehumanization generates negative outcomes operates through multiple interconnected mechanisms. When employees perceive mechanistic dehumanization, they experience threats to fundamental human needs for autonomy, relatedness, and recognition as persons. Individuals subjected to mechanistic dehumanization through objectification, instrumental use, or perceptions that they lack emotional or experiential capacities frequently enter cognitive deconstructive states characterized by impaired clarity of thought, affective numbing, cognitive inflexibility, and a paucity of meaningful mental engagement [25]. Concurrently, such experiences evoke pervasive and intense feelings of sadness and anger [26], reflecting the psychological costs of being treated as an object, a means to an end, or a person lacking feelings. These affective and cognitive disruptions may help explain downstream negative work-related attitudes and behaviors.

Recent studies conducted in organizational settings have elucidated the key antecedents and detrimental outcomes of organizational dehumanization, including the following workplace factors shown to trigger or exacerbate perceptions of being dehumanized: reduced job satisfaction and increased turnover intentions [8,27,28], workplace ostracism [7], decreased affective commitment and heightened psychosomatic strains [22], greater emotional labor and surface acting [29,30], knowledge hiding due to distress [31], increased work-to-family conflict among employees [20]. Organizational animalistic dehumanization makes employees feel that they are treated in animal-like, degrading, or subservient ways, including being overworked, exploited, or expected to remain compliant despite harmful working conditions, thereby generating chronic stress and seriously undermining their psychological and physical well-being [32,33].

In light of these findings, organizational dehumanization emerges as a multifaceted stressor that bridges interpersonal mistreatment, structural dysfunctions, and dehumanizing job designs, ultimately eroding employees’ psychological health, organizational attachment, and proactive contributions. The evidence consistently demonstrates that perceptions of being reduced to mere instruments or expendable livestock not only inflict individual harm through emotional exhaustion, distress, and well-being decline, but also impose hidden costs on organizational performance via disengagement, withdrawal, and counterproductive behaviors. Addressing this phenomenon therefore requires targeted interventions that restore perceptions of fairness, voice, dignity, and meaningful relational support, thereby fostering more humane and sustainable workplaces.

### Dehumanization in higher education context

Contemporary universities are increasingly characterized by organizational arrangements that undermine the human foundations of academic work. While higher education has long been associated with autonomy, critical inquiry, and the pursuit of knowledge as a public good, mounting evidence suggests that academic labor is now governed through marketized, metric-based, and competitive logics that systematically erode academic subjectivity. Within this context, organizational dehumanization is not a peripheral phenomenon but a structural condition of the modern university.

Organizational dehumanization refers to processes through which individuals are treated primarily as functional resources rather than as human beings endowed with dignity, agency, and life narratives. In universities, this process unfolds through interlocking mechanisms of precarity, performance measurement, speed intensification, and the reduction of quality to quantifiable indicators. Together, these mechanisms reshape not only working conditions but also how academics understand themselves, their work, and their place within academic communities. Mason and Megoran [17] identify four interrelated ways in which precarity is dehumanizing: it renders academics invisible within institutions, exposes them to exploitation, denies them academic freedom, and prevents them from constructing a coherent life narrative oriented toward the future. These dimensions move beyond material insecurity and point to a deeper ontological injury. When academics are deprived of continuity, recognition, and autonomy, they are denied key conditions of human flourishing. Precarity thus functions as an organizational mechanism that positions academics as temporary inputs rather than as persons embedded in long-term scholarly projects. This condition is exacerbated by the massification and marketisation of higher education, where entry into and survival within the academic labor market are framed as competitive struggles [34]. Under such conditions, disposability becomes normalized, and insecurity is institutionalized rather than treated as a problem to be resolved. Precarity operates alongside a metric-based organizational culture that defines academic worth through quantification. In marketized universities, quality is increasingly equated with numerical indicators; scores, rankings, and performance metrics; while forms of academic value that resist quantification are dismissed as anecdotal or merely narrative [18]. What counts is what is countable; what cannot be reduced to a metric is excluded from evaluation and, by extension, from institutional recognition.

This metric culture fosters individualist and competitive behaviors that constitute what has been described as the “dark side” of academia [35]. Rather than cultivating collective intellectual communities, universities encourage rivalry, comparison, and strategic self-positioning. Importantly, performance management systems do not simply measure academic reality; they actively construct it. As Kallio et al. [36] argue, by selecting specific indicators and metrics, universities shape what academic work becomes. Over time, institutions quite literally “get what they measure.” The consequences for academic meaning are profound. Research on the publish-or-perish regime illustrates how scholarly productivity becomes a highly evaluative and existentially charged process. Academic careers hinge on performance against targets defined by journal rankings, publication counts, and benchmarked outputs [37]. Failure to meet these expectations can result in reputational damage, stalled promotion, intensified teaching loads, or even job loss. In this environment, research risks losing its intrinsic significance, becoming instead a means of survival within an unforgiving evaluative system.

A further dimension of organizational dehumanization in universities is the contradictory demand to accelerate academic output while coping with the embodied limits of fatigue. Jääskeläinen and Helin [38] describe this tension through the figure of the “Ambivalent Creature,” foregrounding the embodied tensions, vulnerabilities, and resistances involved in academic writing. Within performance-driven academic contexts, academics are also pressured to speed up by publishing more, applying for more grants, and responding faster, while exhaustion simultaneously slows them down. This temporal contradiction is not incidental but structurally embedded in contemporary academic work. Such conditions intensify feelings of alienation and disorientation. Knights and Clarke [39] show that academics frequently experience a disjunction between what they perceive as meaningful work and what institutions reward. While academics may commit themselves to aspirational projects promising security and success, they often report existential anxieties about the lack of deeper significance in their work. Even moments of success, such as publication, offer only temporary relief before the cycle of production resumes. The result is a persistent sense of emptiness, where academic labor is experienced as simultaneously demanding and devoid of lasting meaning.

Organizational dehumanization is further amplified by the psychological consequences of constant evaluation. Scholarly productivity functions as a continuous assessment of worth, rendering academics vulnerable to feelings of inadequacy and fraudulence. Hutchins and Rainbolt [40] show that faculty imposter experiences may be triggered by events such as questioning expertise or legitimacy, scholarly productivity challenges, comparisons with colleagues, and handling successes. For these individuals, failure is not merely professional but deeply personal, triggering intensified self-doubt and overwork. This dynamic reinforces dehumanization by shifting responsibility inward. Structural pressures are internalized as individual shortcomings, prompting academics to work harder to conceal perceived deficiencies. Over time, this pattern increases the risk of burnout and emotional exhaustion, while leaving the organizational conditions that generate these experiences unchallenged.

Beyond individual evaluation, dehumanization also manifests in the erosion of academic communities and sense of place. Nordbäck et al. [19] argue that while global standards of excellence have been widely critiqued, the loss of a shared sense of place within universities has received less attention. Yet academic communities depend on relational belonging and collective identity, both of which are destabilized under neoliberal regimes that privilege mobility, competition, and individual performance. Paradoxically, academic autonomy operates as both a resource and a burden. Bolden et al. [41] show that while flexibility and self-direction are often celebrated as positives of academic work, they are closely linked to work intensification and less visible forms of labor. Academics must continuously decide which tasks to prioritize, often privileging high-status activities such as publishing over undervalued but essential work such as student support, collegial engagement, and administrative or service responsibilities. These dynamics may reproduce unequal burden across roles, career stages, and caring responsibilities, further entrenching dehumanizing distributions of work and reward.

Dehumanization is sustained in part by a decline in empathy as Kteily and Landry [42] note, dehumanization involves denying or overlooking others' humanity. In response, rehumanizing initiatives emphasize the importance of sharing lived experiences and recognizing the broader social factors that shape academic identities [43]. These initiatives highlight that resistance to dehumanization cannot rely solely on individual resilience. Rather, dehumanization must be understood as a collective and organizational process that challenges the logics of metric domination and marketized governance.

Taken together, the growing body of literature suggests that organizational dehumanization in universities arises from the convergence of precarity, metric governance, accelerated work rhythms, and evaluative cultures. Academics are rendered invisible yet overburdened, autonomous yet constrained, celebrated for excellence yet reduced to numbers. These conditions fracture academic identities, erode meaning, and undermine the human foundations of higher education. Understanding universities as potentially dehumanizing organizational contexts is therefore not a rhetorical exaggeration but an analytically grounded conclusion. It shifts attention away from individual coping deficits and toward the organizational structures that may systematically weaken empathy, continuity, and dignity. Addressing dehumanization in universities thus requires not only cultural change but a fundamental rethinking of how academic work is valued, governed, and lived.

### Measures for organizational dehumanization

Organizational dehumanization has been predominantly assessed through self-report scales that focus on employees’ perceptions of being treated as mere instruments or objects by their organization. The most established and widely used measure is the 11-item Organizational Dehumanization Scale developed by Caesens et al. [5]. This unidimensional instrument is grounded in mechanistic dehumanization and assesses employees’ perceptions of being objectified, treated as interchangeable or instrumental resources, and denied subjectivity. The scale has generally shown high reliability (Cronbach’s α > .90) and has been widely used across diverse research designs, occupational sectors, employee groups, and cultural contexts; however, later work has also identified psychometric, conceptual, and practical limitations that motivated the development of shorter validated measures. To address practical constraints in large-scale or longitudinal research, a more efficient 5-item short form was later validated by Lagios et al. [20]. This abbreviated version retains a high correlation with the original 11-item scale while demonstrating comparable reliability and validity, making it particularly suitable when survey length is a concern. While mechanistic dehumanization has dominated the measurement landscape, animalistic organizational dehumanization has received comparatively less scholarly attention until recently. Cheung [32] addressed this gap by developing and validating the Organizational Animalistic Dehumanization Scale through two longitudinal studies. In addition to Cheung’s [32] scale development effort, animalistic organizational dehumanization had been previously addressed by Brison et al. [44]. Their study introduced a brief five-item measure designed to capture employees’ perceptions of being treated in animal-like, degrading, or uncivilized ways in the workplace. Empirical results showed that animalistic organizational dehumanization was associated with mechanistic dehumanization and with relevant employee outcomes, including job satisfaction, turnover intentions, and in-role performance. However, as an early and exploratory attempt, the study provided limited information regarding the item development and selection process, which constrains the assessment of its conceptual grounding and content validity. As such, while Brison et al.’s [44] work represents an important precursor in the measurement of animalistic dehumanization at work, more recent efforts offer a more systematic and theoretically transparent operationalization of the construct.

This study aims to address gaps in the literature by developing and validating a new scale informed by the conceptualization and measurement tradition of organizational dehumanization introduced by Caesens et al. [5]. The scale consists of two subscales: Devaluation, capturing perceptions of not being valued, being ignored, restricted emotional expression, and injustice-related experiences; and Instrumentality, capturing instrumentalization, objectification, machine-like treatment, and replaceability perceptions. This two-dimensional structure enables a more comprehensive measurement of mechanistic dehumanization by simultaneously addressing both its emotional/value-based and functional/instrumental aspects, compared to the predominantly unidimensional approaches in the existing literature. The scale was applied to academics in Türkiye, allowing an examination of how dehumanization is experienced within the unique dynamics of higher education institutions. Several items explicitly capture academics’ perceptions of being treated as replaceable resources and instrumental means for institutional goals, reflecting the distinctive performance and evaluation dynamics of higher education.

## Method

### Participants

In this study, data were collected from two independent samples of academics to examine the psychometric properties of the newly developed Multidimensional Organizational Dehumanization Scale. Evidence based on the internal structure of the scale was first examined through exploratory factor analysis (EFA) using the first sample, which consisted of 318 academics. The factor structure identified in the EFA was then evaluated through confirmatory factor analysis (CFA) using a second, independent sample consisting of 263 academics with broadly comparable characteristics. Overall, the study included 581 participants across the two samples.

The EFA sample was used to analyze the factor structure of the scale and to conduct item analyses. The CFA sample was used to test the model fit of the structure identified through EFA using an independent dataset and to calculate internal consistency estimates for the retained scale dimensions.

### Item development and data collection procedure

Prior to the development of the item pool, the relevant literature on organizational dehumanization was reviewed, and an initial set of 38 statements aimed at measuring academics' perceptions of organizational dehumanization was generated. The item pool was informed by the conceptualization and measurement tradition of organizational dehumanization, particularly the work of Caesens et al. [5], but the items were developed for the present study and the higher education context.

Before data collection commenced, the necessary ethical approval was obtained from the Non-Interventional Clinical Research Ethics Committee of Biruni University. Following ethical approval, the data collection process was initiated.

Data were collected prospectively using online survey platforms across two distinct recruitment phases corresponding to the exploratory and confirmatory analyses. For the Exploratory Factor Analysis (EFA), data were collected via Jotform between 14 June 2023 and 16 July 2023, during which responses were obtained from 318 academics. For the Confirmatory Factor Analysis (CFA), an independent sample was recruited using Google Forms between 12 August 2023 and 25 September 2023, yielding responses from 263 academics.

Participation in both phases of the study was voluntary. Prior to accessing the questionnaire, all participants were provided with detailed information regarding the purpose of the study, data confidentiality, and their right to withdraw at any time without penalty. Informed consent was obtained electronically through the survey platforms, whereby participants were required to actively indicate their consent before proceeding with the questionnaire. Only individuals who provided informed consent were able to participate. All participants were adults; therefore, no parental or guardian consent was required, and the requirement for informed consent was not waived by the ethics committee.

Participants responded to each item using a 5-point Likert-type scale ranging from 1 (strongly disagree) to 5 (strongly agree), indicating the extent to which each statement reflected their feelings, thoughts, and experiences related to organizational dehumanization. Negatively worded items were reverse coded prior to analysis.

Sample items included “No matter what I do, I feel that I do not receive the recognition I deserve in my institution,” representing the Devaluation dimension, and “My institution sees me as a tool to be used for its own purposes,” representing the Instrumentality dimension.

### Data analysis

Item analyses, exploratory factor analysis, and internal consistency analyses were conducted using IBM SPSS Statistics Version 25.0. Confirmatory factor analysis was performed using LISREL Version 8.80 with maximum likelihood estimation. Prior to the main analyses, the data were organized and screened for accuracy. A pilot study was conducted to assess the clarity and comprehensibility of the scale items for participants. The preliminary administration was carried out face-to-face with a group of 20 individuals who met the inclusion criteria for the study sample.

Using the EFA sample of 318 academic staff members, item analyses and exploratory factor analysis were conducted. To determine the adequacy of the sample size for factor analysis, the suitability of the dataset was examined and found to be consistent with the recommendations of Tabachnick and Fidell [45].

To obtain evidence based on the internal structure of the scale, an Exploratory Factor Analysis (EFA) was performed using principal axis factoring with varimax rotation. Principal axis factoring was preferred as it focuses on shared variance and is recommended for latent construct identification in scale development studies. In accordance with the criteria suggested by Çokluk et al. [46], factor loadings of at least .30 were retained. Cronbach’s alpha coefficients were calculated to assess internal consistency.

Prior to calculating internal consistency coefficients, two item analysis methods were employed: (a) item–total score correlations, and (b) item discrimination analysis based on differences between upper and lower group means. Scale scores obtained from the academics’ responses were ranked from highest to lowest. Among the 318 participants, the lowest-scoring 86 individuals were classified as the lower group, while the highest-scoring 86 individuals were classified as the upper group. Based on this classification, the differences between the mean scores of the upper and lower groups for each item were examined using independent samples t-tests. In addition, to evaluate the model fit of the factor structure identified through EFA, a Confirmatory Factor Analysis (CFA) was conducted using the independent CFA sample.

## Results

The findings are presented below in relation to item analyses, exploratory factor analysis, confirmatory factor analysis, convergent validity, discriminant validity, and reliability. As shown in Table 1, the factor loadings of the retained items ranged from  .496 to  .795, exceeding the minimum criterion of  .30. The corrected item–total correlation coefficients also indicated acceptable item–scale associations. Comparisons of the mean item scores between the upper and lower 27% groups revealed statistically significant differences for all items (p < .001), indicating satisfactory item discrimination.

**Table 1:** Factor loadings, item–total correlation coefficients, and t values.

Items	Factor 1	Factor 2	*r*	*t*
I4	.795		.741	−17.488
I5	.774		.797	−19.790
I8	.738		.678	−13.986
I3	.713		−.550	9.498
I9	.692		.805	−21.743
I19	.691		.679	−13.768
I7	.682		.739	−17.429
I13	.681		.722	−17.051
I23	.677		.744	−20.020
I22	.672		.773	−17.610
I24	.667		.725	−17.981
I1	.666		.721	−16.203
I20	.648		.620	−12.978
I6	.640		.789	−22.011
I16	.637		.744	−16.269
I21	.630		.644	−13.704
I11	.620		.717	−14.680
I14	.593		.702	−14.413
I10	.573		.527	−10.067
I25	.558		.709	−14.509
I2	.496		.503	−10.041
I28		.779	.644	−13.225
I29		.775	.676	−14.688
I30		.774	.877	−29.377
I34		.757	.814	−23.732
I27		.753	.822	−21.380
I38		.745	.779	−18.521
I31		.740	.878	−35.323
I37		.738	.776	−20.063
I33		.733	.863	−28.573
I32		.713	.884	−29.913
I35		.689	.862	−29.200
I36		.675	.644	−12.312
I26		.623	.455	−6.954
Eigenvalue: Factor 1 = 18.267, Factor 2 = 2.335 Explained variance (%): Factor 1 = 53.73, Factor 2 = 6.87				

***Note.*** *r = corrected item–total correlation coefficient; t = independent-samples t-test statistic comparing the upper 27% and lower 27% groups for item discrimination; Factor 1 and Factor 2 = factor loadings obtained from exploratory factor analysis using principal axis factoring with varimax rotation. Blank cells indicate that the item did not load meaningfully on the corresponding factor. Eigenvalues and explained variance percentages are reported for each factor at the bottom of the table.*

### Exploratory factor analysis

An Exploratory Factor Analysis (EFA) was conducted to obtain evidence based on the internal structure of the Multidimensional Organizational Dehumanization Scale. After the initial item analyses and item-retention procedures, the suitability of the final 34-item dataset for factor analysis was evaluated using the Kaiser–Meyer–Olkin (KMO) measure of sampling adequacy and Bartlett’s test of sphericity. The KMO value was found to be .964, and Bartlett’s test of sphericity was statistically significant, χ²(561) = 9,864.93, p < .001. A KMO value greater than .60 and a significant Bartlett’s test indicate that the data are suitable for factor analysis [45].

After confirming the suitability of the data for factor analysis, EFA was performed without specifying the number of factors in advance, including the reverse-coded items. When eigenvalues greater than 1.0 were considered, the scale initially appeared to have a three-factor structure; however, examination of the scree plot and the interpretability of the factor solution supported the retention of a two-factor structure.

In determining the factor structure, items were retained if they had factor loadings of at least .30, and if the difference between cross-loadings on two factors was at least .10. Based on these criteria, four items that did not meet the requirements were removed from the scale. The remaining two factors, extracted using varimax rotation, explained a total of 60.59% of the variance. The first factor, labeled “Devaluation,” accounted for 53.73% of the variance, whereas the second factor, labeled “Instrumentality,” accounted for 6.87% of the variance.

These results indicate that the scale yielded an interpretable two-factor structure of academics’ perceptions of organizational dehumanization. The model fit of the resulting factor structure and the obtained values were further examined using Confirmatory Factor Analysis (CFA).

### Confirmatory factor analysis

During the Confirmatory Factor Analysis (CFA), multiple goodness-of-fit indices were evaluated, including the chi-square goodness-of-fit statistic (χ²), Normed Fit Index (NFI), Relative Fit Index (RFI), Comparative Fit Index (CFI), Goodness-of-Fit Index (GFI), Adjusted Goodness-of-Fit Index (AGFI), Standardized Root Mean Square Residual (SRMR), Incremental Fit Index (IFI), and the Root Mean Square Error of Approximation (RMSEA) [47,48]. The fit indices obtained from the CFA are presented in Table 2.

**Table 2:** Goodness-of-fit indices.

*X* ^*2*^	*X* ^*2*^ */df*	*p*	NFI	RFI	CFI	GFI	AGFI	IFI	RMSEA	SRMR
1196.15	2.28	< .001	.97	.97	.99	.78	.75	.99	.074	.045

***Note.*** *χ² = chi-square goodness-of-fit statistic; df = degrees of freedom; χ²/df = chi-square divided by degrees of freedom; p = significance level of the chi-square test; NFI = Normed Fit Index; RFI = Relative Fit Index; CFI = Comparative Fit Index; GFI = Goodness-of-Fit Index; AGFI = Adjusted Goodness-of-Fit Index; IFI = Incremental Fit Index; RMSEA = Root Mean Square Error of Approximation; SRMR = Standardized Root Mean Square Residual.*

In evaluating model fit, the ratio of the chi-square value to the degrees of freedom (*χ²/df*) was also considered as a descriptive fit indicator [49]. Because fit-index cutoff values should not be treated as rigid universal rules, the fit indices were interpreted jointly [48,50]. As shown in Table 2, the chi-square statistic was statistically significant (χ² = 1,196.15, *df* = 524, χ²/df = 2.28, *p* < .001).

In this study, the Goodness-of-Fit Index (GFI) was found to be .78, and the Adjusted Goodness-of-Fit Index (AGFI) was .75. With respect to other fit indices, NFI =  .97, RFI =  .97, CFI =  .99, IFI =  .99, RMSEA =  .074, and SRMR =  .045. The incremental fit indices indicated strong fit, while RMSEA and SRMR were within commonly used acceptable ranges [48,50]. However, because GFI and AGFI were below commonly recommended cutoff values, the results were interpreted as indicating acceptable overall fit rather than uniformly high model fit. The fit indices of the 34-item scale were evaluated together with item factor loadings, t values, error variances, and explained variances (R²). The results obtained from these analyses are presented in Table 3.

**Table 3:** Item factor loadings, t values, error variances, and explained variances.

Items	*λ*	*t*	Error variance	*R²*
I1	.79	15.36	.37	.63
I2	.47	8.04	.77	.23
I3	−.70	−12.84	.51	.49
I4	.84	16.62	.30	.70
I5	.85	17.02	.28	.72
I6	.77	14.66	.41	.59
I7	.78	14.98	.39	.61
I8	.81	15.84	.34	.66
I9	.78	15.07	.39	.61
I10	.60	10.53	.64	.36
I11	.76	14.45	.42	.58
I12	.56	9.84	.68	.32
I13	.70	12.81	.52	.48
I14	.64	11.58	.58	.42
I15	.78	14.91	.40	.60
I16	.75	14.22	.43	.57
I17	.61	10.86	.62	.38
I18	.62	11.12	.61	.39
I19	.84	16.82	.29	.71
I20	.82	16.24	.32	.68
I21	.80	15.52	.36	.64
I22	.74	12.83	.46	.54
I23	.46	7.80	.79	.21
I24	.85	17.02	.28	.72
I25	.67	12.27	.55	.45
I26	.72	13.56	.47	.53
I27	.92	19.48	.16	.84
I28	.92	19.56	.15	.85
I29	.94	20.42	.11	.89
I30	.91	19.20	.17	.83
I31	.85	17.02	.28	.72
I32	.87	17.78	.24	.76
I33	.82	16.42	.31	.69
I34	.90	18.80	.19	.81

***Note.*** *λ = standardized factor loading obtained from confirmatory factor analysis (CFA); t = t value for the factor loading; Error variance = unexplained variance for each item; R² = squared multiple correlation, representing the proportion of item variance explained by the latent factor. Absolute t values greater than 1.96 indicate statistical significance at p < .05. Negative factor loadings reflect the scoring direction of the corresponding item.*

### Convergent validity

Convergent validity evidence was examined within the broader framework of validity evidence based on relationships among variables [51]. For this purpose, composite reliability (CR) and average variance extracted (AVE) values were calculated. In addition, standardized factor loadings, t values, and item reliability estimates were evaluated as indicators of the measurement quality of the items. Item reliability indicates the extent to which an item is consistent with the construct being measured. When a large proportion of an item’s variance is explained by the underlying construct, the measurement instrument can be considered reliable.

Standardized factor loadings and statistically significant t values were considered when evaluating item-level evidence for convergent validity [52,53]. The t values of all items were statistically significant. Although most standardized factor loadings were at or above the commonly used  .50 level, two items had slightly lower loadings. In addition, item reliability values varied across items, indicating that convergent validity evidence should be interpreted together with CR and AVE rather than on the basis of item reliability alone.

Composite reliability reflects the internal consistency and reliability of the subdimensions within a measurement instrument and is considered one of the fundamental criteria for evaluating measurement models. A composite reliability value of .70 or higher is regarded as acceptable. In the present study, composite reliability values were high for both subdimensions, supporting the internal consistency of the two-factor measurement model [53,54].

The AVE value, another indicator of convergent validity, is expected to be .50 or higher [53,54]. While the Instrumentality subdimension met this criterion, the Devaluation subdimension yielded an AVE value below the recommended threshold. Therefore, convergent validity evidence was stronger for the Instrumentality subdimension than for the Devaluation subdimension.

Finally, the relationships between the two subdimensions of the Multidimensional Organizational Dehumanization Scale were found to be statistically significant at the .01 level, with moderate correlations observed between the subdimensions. This finding indicates that the two subdimensions are related but not redundant.

### Discriminant validity

Discriminant validity assesses the extent to which a measurement instrument is able to distinguish between different constructs. In the present study, discriminant validity was evaluated using the criterion proposed by Fornell and Larcker [54]. According to this approach, the square of the correlation between two constructs is compared with the average variance extracted (AVE) values of each construct. Discriminant validity is considered to be established when the AVE values of both constructs are greater than the squared correlation between them. Based on the comparison between the calculated squared correlation and the AVE values, the results indicated that discriminant validity was achieved.

### Reliability

The reliability of the scale was evaluated using Cronbach’s alpha coefficient based on internal consistency. Cronbach’s alpha values were calculated for each factor and for the overall scale. The obtained coefficients ranged from .93 to .96.

Specifically, the Cronbach’s alpha coefficient was .93 for the first factor, .96 for the second factor, and .96 for the overall scale. These results indicate that the scale demonstrated high internal consistency reliability.

## Discussion

In this study, a Multidimensional Organizational Dehumanization Scale was developed and validated among academics. To determine the items to include in the scale, analyses of item–total score correlations and differences between upper and lower groups were conducted. All parameters obtained were found to be statistically significant. The mean responses of participants in the upper and lower groups differed significantly for all items. These findings indicate that each item in the scale possesses the necessary psychometric properties and demonstrates adequate item discrimination.

To establish the construct validity of the dehumanization scale, both Exploratory Factor Analysis (EFA) and Confirmatory Factor Analysis (CFA) were conducted. The results of the EFA revealed a two-factor structure, with the two factors together explaining 60.59% of the total variance. The first factor, labeled Devaluation, accounted for 53.73% of the variance, whereas the second factor, labeled Instrumentality, explained 6.87% of the variance. Following varimax rotation, four items that did not meet the item-retention criteria were removed, resulting in a final 34-item scale.

The factor structure and model fit obtained through EFA were subsequently tested using CFA. The fit indices were calculated as χ² = 1,196.15, χ²/df = 2.28, NFI = .97, RFI = .97, CFI = .99, GFI = .78, AGFI = .75, IFI = .99, SRMR = .045, and RMSEA = .074. Interpreted jointly, these values indicate acceptable overall fit between the hypothesized model and the observed data, although GFI and AGFI were below commonly recommended cutoff values [55]. Taken together, the results of the EFA and CFA demonstrate that the two-factor, 34-item model is both theoretically and statistically sound, providing strong evidence for the structural validity of the scale.

Convergent validity of the scale was evaluated using item reliability, composite reliability, and average variance extracted (AVE) values. The t values were statistically significant, and the factor loadings generally supported item-level evidence for convergent validity, although item reliability values varied across items. Composite reliability values supported the internal consistency of the two subdimensions. However, the AVE value for one subdimension fell below the recommended threshold. The internal consistency reliability coefficient of the overall scale was found to be .96, indicating a high level of reliability.

Overall, the analyses provide evidence that the developed scale possesses acceptable psychometric properties. The scale can be used to assess perceptions of organizational dehumanization among academics and contributes to the literature by providing a psychometrically sound two-dimensional measurement instrument for examining dehumanization in academic contexts. The findings further suggest that organizational dehumanization can be assessed through multiple dimensions and that the scale is suitable for evaluating whether academics perceive dehumanizing attitudes toward themselves within their institutions.

### Limitations and future research

Several limitations should be considered when interpreting the findings of this study. First, although the scale was developed and validated using two independent samples, the data were collected through online self-report surveys from academics. As with most self-report designs, responses may be influenced by common method effects or response styles.

Second, although the CFA results indicated an acceptable overall model fit, with several indices suggesting strong fit, some fit indices, particularly GFI and AGFI, were relatively lower. In addition, convergent validity evidence showed that AVE for one subdimension did not meet the conventional threshold. Future studies should further examine the measurement model using alternative estimation strategies and additional samples, and consider targeted item refinement to improve the subdimension’s convergent validity while preserving conceptual coverage.

Finally, while internal consistency reliability estimates were very high, additional forms of reliability evidence remain important. Future research should report test–retest reliability explicitly, including the time interval between administrations, and evaluate longitudinal stability, particularly because dehumanization perceptions may fluctuate with organizational events, workload cycles, and performance evaluation periods in universities.

Building on these limitations, future research should also investigate criterion-related validity evidence by examining relationships between the scale scores and theoretically relevant outcomes in higher education such as burnout, job satisfaction, turnover intentions, organizational commitment, psychological well-being, as well as potential antecedents such as perceived organizational support, procedural justice, leadership practices, and metric/managerial pressures.

In addition, future validation studies may provide further psychometric evidence by reporting alternative reliability estimates such as McDonald’s omega, examining item functioning using item response theory when sample size permits, and testing measurement invariance across key demographic groups such as gender, academic rank, institution type, and disciplinary field. Such analyses would strengthen the generalizability of the scale and clarify whether the two-dimensional structure operates equivalently across different groups of academics.

## Conclusion

This study developed and provided validation evidence for a new Multidimensional Organizational Dehumanization Scale for academics designed to assess perceptions of dehumanization within higher education institutions. Item analyses indicated that all retained items showed adequate discrimination, with statistically significant differences between upper and lower groups (p < .001) and satisfactory item–total correlations. Exploratory factor analysis supported a two-factor structure, and the resulting model was further evaluated through confirmatory factor analysis using an independent sample. Overall, the evidence supports the structural validity of the 34-item scale comprising two subdimensions: Devaluation and Instrumentality.

Reliability findings demonstrated high internal consistency for both subscales and the total score. Additional validity evidence was provided through convergent and discriminant validity analyses, although one subdimension’s AVE did not meet the conventional threshold, indicating that further validation and refinement may be beneficial. Despite this limitation, the overall pattern of results suggests that the scale provides a psychometrically acceptable and practically useful multidimensional measure of organizational dehumanization perceptions among academics.

In sum, the scale constitutes a valuable contribution to the emerging literature on organizational dehumanization by offering a multidimensional operationalization developed for and evaluated within a higher education sample. It can be used in future research to examine antecedents and consequences of dehumanization in universities and to support organizational diagnostics and interventions aimed at promoting more humane academic workplaces.

### Institutional ethical board approval and informed consent

The study was approved by the Non-Interventional Clinical Research Ethics Committee of Biruni University (decision number: 2023/78–07, meeting number: 78, date of approval: 21 February 2023). All methods were carried out in accordance with the relevant guidelines and regulations, including the ethical standards established in the Declaration of Helsinki. Informed consent was obtained from all participants prior to data collection. All data were collected anonymously and analyzed solely for the purpose of examining the psychometric properties of the developed instrument.

## Supporting information

S1 Appendix Scale items originally developed and validated in Turkish with English equivalents. The original Turkish items of the Multidimensional Organizational Dehumanization Scale, which was developed and validated in Turkish, are provided in S1 Appendix together with English equivalents prepared for international readership.
(DOCX)
