# Supplementary material for: Development and validation of a multidimensional organizational dehumanization scale: Evidence from higher education
Source: PLoS One. 2026 Jul 6;21(7):e0351254. doi: 10.1371/journal.pone.0351254 (PMC13336461; doi:10.1371/journal.pone.0351254)
Supplement: S1 Appendix — The original Turkish items of the Multidimensional Organizational Dehumanization Scale, which was developed and validated in Turkish, are provided in S1 Appendix together with English equivalents prepared for international readership. (DOCX) [file pone.0351254.s001.docx]

**Appendix**

**Appendix Table 1.** The equivalents of the developed and validated Multidimensional Organizational Dehumanization Scale in the English language.

| Item Number | Below are statements related to experiences of dehumanization. Please indicate the extent to which you agree with each statement by marking the appropriate option. | Strongly Agree | Agree | Neutral | Disagree | Strongly Disagree |
| --- | --- | --- | --- | --- | --- | --- |
| 1 | No matter what I do, I feel that I do not receive the recognition I deserve in my institution. | **5** | **4** | **3** | **2** | **1** |
| 2 | When things are running smoothly in my institution, people do not communicate with one another. | **5** | **4** | **3** | **2** | **1** |
| 3 | I feel comfortable talking about my problems in my institution. | **5** | **4** | **3** | **2** | **1** |
| 4 | My institution takes my ideas seriously. | **5** | **4** | **3** | **2** | **1** |
| 5 | My institution does not value me. | **5** | **4** | **3** | **2** | **1** |
| 6 | In my institution, people are valued only as long as tasks are completed. | **5** | **4** | **3** | **2** | **1** |
| 7 | In my institution, I cannot express my genuine feelings. | **5** | **4** | **3** | **2** | **1** |
| 8 | The work I do is appreciated in my institution. | **5** | **4** | **3** | **2** | **1** |
| 9 | In my institution, I am given attention only when I am needed. | **5** | **4** | **3** | **2** | **1** |
| 10 | My institution makes me feel valued on special occasions (e.g., birthdays, holidays, becoming a parent, promotion). | **5** | **4** | **3** | **2** | **1** |
| 11 | I feel that my institution does not fulfill its responsibilities toward me. | **5** | **4** | **3** | **2** | **1** |
| 12 | I feel that my institution does not value my opinions. | **5** | **4** | **3** | **2** | **1** |
| 13 | I feel that my personal problems are not taken seriously in my institution. | **5** | **4** | **3** | **2** | **1** |
| 14 | My institution treats me in an impolite or disrespectful manner. | **5** | **4** | **3** | **2** | **1** |
| 15 | My institution makes me feel that I am at least as competent as other employees. | **5** | **4** | **3** | **2** | **1** |
| 16 | My institution supports my participation in meetings related to my area of work. | **5** | **4** | **3** | **2** | **1** |
| 17 | My institution supports my participation in activities related to my professional development (e.g., courses, training). | **5** | **4** | **3** | **2** | **1** |
| 18 | I feel that my institution ignores me. | **5** | **4** | **3** | **2** | **1** |
| 19 | My institution treats me and other employees fairly. | **5** | **4** | **3** | **2** | **1** |
| 20 | My institution treats me and other employees equally. | **5** | **4** | **3** | **2** | **1** |
| 21 | My institution withholds work-related information from me. | **5** | **4** | **3** | **2** | **1** |
| 22 | If it would benefit the institution financially, it would not hesitate to replace its staff. | **5** | **4** | **3** | **2** | **1** |
| 23 | My institution sees me as a tool to be used for its own purposes. | **5** | **4** | **3** | **2** | **1** |
| 24 | My institution views me as an instrument devoted to the institution’s success. | **5** | **4** | **3** | **2** | **1** |
| 25 | The only thing that matters to my institution is how much I contribute to it. | **5** | **4** | **3** | **2** | **1** |
| 26 | My institution treats me as if I were a robot. | **5** | **4** | **3** | **2** | **1** |
| 27 | My institution sees me as a number rather than as a person. | **5** | **4** | **3** | **2** | **1** |
| 28 | My institution treats me as if I were an object. | **5** | **4** | **3** | **2** | **1** |
| 29 | My institution sees me as a machine rather than as an academic. | **5** | **4** | **3** | **2** | **1** |
| 30 | If my job could be done by a machine or a robot, my institution would not hesitate to replace me. | **5** | **4** | **3** | **2** | **1** |
| 31 | My institution treats me as if I have no emotions. | **5** | **4** | **3** | **2** | **1** |
| 32 | In my institution, I am assigned work as if I would never get tired. | **5** | **4** | **3** | **2** | **1** |
| 33 | My institution sees me not as an academic, but merely as someone who can perform any task. | **5** | **4** | **3** | **2** | **1** |
| 34 | My institution has irrational or unreasonable expectations of me. | **5** | **4** | **3** | **2** | **1** |

**Appendix Table 2.** The original items of the developed and validated Multidimensional Organizational Dehumanization Scale in the Turkish language.

| Item Number | Aşağıda İnsandışılaştırmaya yönelik ifadeler yer almaktadır. Lütfen bu ifadelerin her birine ne ölçüde katıldığınızı ilgili parantezi X ile işaretleyerek belirtiniz. | Strongly Agree | Agree | Neutral | Disagree | Strongly Disagree |
| --- | --- | --- | --- | --- | --- | --- |
| 1 | Ne yaparsam yapayım çalıştığım kurumda hak ettiğim değeri alamam. | **5** | **4** | **3** | **2** | **1** |
| 2 | Çalıştığım kurumda işler yolundaysa kimse birbiriyle iletişim kurmaz. | **5** | **4** | **3** | **2** | **1** |
| 3 | Çalıştığım kurumda rahatlıkla bir problemimden bahsedebilirim. | **5** | **4** | **3** | **2** | **1** |
| 4 | Çalıştığım kurum fikirlerimi önemser. | **5** | **4** | **3** | **2** | **1** |
| 5 | Çalıştığım kurum bana değer vermez. | **5** | **4** | **3** | **2** | **1** |
| 6 | Çalıştığım kurumda sadece işlerin hallolması için değer verilir. | **5** | **4** | **3** | **2** | **1** |
| 7 | Çalıştığım kurumda gerçek duygularımı ifade etmem mümkün değildir. | **5** | **4** | **3** | **2** | **1** |
| 8 | Çalıştığım kurumda yaptığım işe değer verilir. | **5** | **4** | **3** | **2** | **1** |
| 9 | Çalıştığım kurumda benimle sadece ihtiyaç olduğunda ilgilenilir. | **5** | **4** | **3** | **2** | **1** |
| 10 | Çalıştığım kurum; özel günlerde (doğum günü, bayram, anne veya baba olma, terfi etme vb.) beni değerli hissettirir. | **5** | **4** | **3** | **2** | **1** |
| 11 | Çalıştığım kurumun benimle ilgili sorumluluklarını yerine getirmediğini düşünürüm. | **5** | **4** | **3** | **2** | **1** |
| 12 | Çalıştığım kurumun benim fikirlerime önem vermediğini düşünürüm. | **5** | **4** | **3** | **2** | **1** |
| 13 | Çalıştığım kurumda şahsi problemlerimi umursamadıklarını düşünürüm. | **5** | **4** | **3** | **2** | **1** |
| 14 | Çalıştığım kurum bana nezaketsiz davranmaktadır. | **5** | **4** | **3** | **2** | **1** |
| 15 | Çalıştığım kurum bana bir çalışanın en az diğerleri kadar iyi olduğunu hissettirir. | **5** | **4** | **3** | **2** | **1** |
| 16 | Çalıştığım kurum çalışma alanımla ilgili yapılan toplantılara katılımımı destekler/sağlar. | **5** | **4** | **3** | **2** | **1** |
| 17 | Çalıştığım kurum gelişimimle ilgi etkinliklere (kurs, eğitim vb.) katılımımı destekler. | **5** | **4** | **3** | **2** | **1** |
| 18 | Çalıştığım kurumun beni yok saydığını düşünürüm. | **5** | **4** | **3** | **2** | **1** |
| 19 | Çalıştığım kurum diğer çalışanlara ve bana adil davranmaktadır. | **5** | **4** | **3** | **2** | **1** |
| 20 | Çalıştığım kurum diğer çalışanlara ve bana eşit davranmaktadır. | **5** | **4** | **3** | **2** | **1** |
| 21 | Çalıştığım kurum işimle ilgili bilgileri benden gizlemektedir. | **5** | **4** | **3** | **2** | **1** |
| 22 | Çalıştığım kurum daha fazla kâr etmesini sağlayacaksa personel değişikliği yapmaktan çekinmez. | **5** | **4** | **3** | **2** | **1** |
| 23 | Çalıştığım kurum beni kendi amaçları için kullanacağı bir araç olarak görmektedir. | **5** | **4** | **3** | **2** | **1** |
| 24 | Çalıştığım kurum beni kurum başarısına adanmış bir araç olarak görmektedir. | **5** | **4** | **3** | **2** | **1** |
| 25 | Çalıştığım kurum için önemli olan tek şey, ona ne kadar katkıda bulunacağımdır. | **5** | **4** | **3** | **2** | **1** |
| 26 | Çalıştığım kurum bana bir robotmuşum gibi davranmaktadır. | **5** | **4** | **3** | **2** | **1** |
| 27 | Çalıştığım kurum beni bir sayı olarak görmektedir. | **5** | **4** | **3** | **2** | **1** |
| 28 | Çalıştığım kurum bana bir nesneymişim gibi davranmaktadır. | **5** | **4** | **3** | **2** | **1** |
| 29 | Çalıştığım kurum beni bir akademisyen olarak değil, makine olarak görmektedir. | **5** | **4** | **3** | **2** | **1** |
| 30 | Eğer işimi bir makine veya robot yapabilseydi, kurumum beni bu alet ile değiştirmekte tereddüt etmezdi. | **5** | **4** | **3** | **2** | **1** |
| 31 | Çalıştığım kurum bana duygularım yokmuş gibi davranır. | **5** | **4** | **3** | **2** | **1** |
| 32 | Çalıştığım kurumda bana hiç yorulmayacakmışım gibi iş verilir. | **5** | **4** | **3** | **2** | **1** |
| 33 | Çalıştığım kurum beni bir akademisyen olarak değil de yalnızca her işi yapabilecek bir kişi olarak görmektedir. | **5** | **4** | **3** | **2** | **1** |
| 34 | Çalıştığım kurum benden rasyonel olmayan beklentiler içerisindedir. | **5** | **4** | **3** | **2** | **1** |
